# Exploring the Potential Roles of Oxidative Stress–Related Genes in Thyroid Eye Disease and Graves’ Disease

**DOI:** 10.1155/joph/9124511

**Published:** 2026-02-13

**Authors:** Weili Zhang, Qinying Huang, Jinying Li

**Affiliations:** ^1^ Department of Ophthalmology, Peking University Shenzhen Hospital, Shenzhen, Guangdong, 518036, China, pkuszh.com

**Keywords:** differentially expressed genes (DEGs), Gene Expression Omnibus database (GEO database), Graves’disease (GD), oxidative stress, thyroid eye disease (TED)

## Abstract

**Purpose:**

To reveal the significance of oxidative stress–related genes in the pathogenesis of thyroid eye disease (TED) and Graves’ disease (GD) using a bioinformatics approach.

**Methods:**

Utilizing datasets from the GEO database, we used the “limma” package to detect differentially expressed genes (DEGs). Oxidative stress–related DEGs related to TED and GD were identified through cross‐referencing with the GeneCards database. We used a variety of methods, such as enrichment analyses, LASSO, RF techniques, PPI network analysis, and the CIBERSORT algorithm.

**Results:**

We identified 22 oxidative stress–related DEGs related to TED and GD, primarily involved in miRNA transcription and regulation. Hub genes (DUSP1, EGR1, FOS, and JUNB) were linked to immune cells and were identified as potential diagnostic biomarkers. The developed nomogram model exhibited satisfactory calibration.

**Conclusion:**

This computational study sheds light on the molecular pathways underlying TED and GD, proposing candidate biomarkers and therapeutic targets. However, these findings are preliminary and require further experimental validation (e.g., qPCR, western blot, and IHC) in patient tissues before their clinical utility can be assessed.

## 1. Introduction

Thyroid eye disease (TED) and Graves’ disease (GD) are autoimmune disorders that significantly impair patients’ quality of life [1–3]. TED, which involves inflammation and swelling of the ocular muscles and surrounding tissues, develops in roughly 25%–50% of GD patients, the most frequent cause of hyperthyroidism [4–6]. The rising prevalence of these conditions has escalated healthcare expenditures, imposing a considerable economic burden on both patients and society. Current clinical management of TED and GD relies primarily on corticosteroids, immunosuppressants, and surgical procedures [7, 8]. These treatments, however, often produce inconsistent outcomes and are linked to substantial adverse effects. Moreover, the absence of specific biomarkers for early diagnosis and an incomplete understanding of the underlying pathophysiology impede the development of targeted therapies. Consequently, novel strategies are urgently required to enhance diagnostic accuracy and therapeutic efficacy for TED and GD.

TED and GD are characterized by dysregulated thyroid function and immune cell infiltration into the orbital tissues, which drive inflammation and tissue remodeling [9]. Oxidative stress, which results from an imbalance between antioxidant defenses and reactive oxygen species (ROS) production, plays a central role in their pathophysiology [10]. Elevated ROS levels induce cellular damage, promote inflammation, and exacerbate tissue injury, thereby contributing to the clinical manifestations of both disorders [11].

Recent studies underscore the critical role of oxidative stress–related genes in the progression of TED and GD [12]. Differential expression patterns are observed in genes governing antioxidant responses, including superoxide dismutases (SODs) and glutathione peroxidases (GPXs), in affected patients [13]. Disruptions in the expression of these genes correlate with the severity of both ocular manifestations and thyroid dysfunction, suggesting their potential as biomarkers for monitoring disease progression. Despite compelling evidence linking oxidative stress to TED and GD, the underlying molecular mechanisms remain incompletely understood. Existing research often lacks comprehensive analyses of the immune microenvironment and the intricate interplay between oxidative stress and immune cell infiltration. Furthermore, the specificity of available biomarkers is limited, highlighting the need to elucidate the involved molecular pathways. Addressing these gaps may enable novel therapeutic strategies to mitigate oxidative stress and its detrimental effects in TED and GD.

This study investigates the molecular basis of TED and GD by analyzing differentially expressed genes (DEGs) related to oxidative stress. We aim to identify potential biomarkers and therapeutic targets through the bioinformatic analysis of transcriptomic data. Relevant datasets will be obtained from the Gene Expression Omnibus (GEO) database, followed by enrichment analysis to elucidate the associated biological pathways. Key diagnostic genes will be identified using robust statistical methods, including random forest (RF) and least absolute shrinkage and selection operator (LASSO) regression analysis. A protein–protein interaction (PPI) network will then be constructed to highlight hub DEGs. This research may provide a more comprehensive understanding of the immune microenvironment in TED and GD. The integrative approach is intended to yield insights into disease pathophysiology and inform the development of future therapeutic strategies.

## 2. Materials and Methods

### 2.1. Data Collection

We obtained the datasets related to TED and GD from the GEO (https://www.ncbi.nlm.nih.gov/geo/) database using the search terms “Thyroid eye disease” and “Graves’ disease.” Oxidative stress genes were retrieved from GeneCards (https://www.genecards.org/, accessed 22 Oct 2024), and all downloaded datasets were derived from human samples with a minimum of 10 samples each. The GSE58331 and GSE71956 datasets were downloaded from platforms GPL570 and GPL10558, respectively. GSE58331 consists of anterior orbit tissues from 27 TED patients and 22 healthy controls, whereas GSE71956 contains total RNA extracted from T cells of 31 GD patients and 18 healthy controls.

### 2.2. Identification of DEGs Related to TED and GD

DEGs in the GSE58331 and GSE71956 datasets were identified using the “limma” package in R, applying thresholds of |log2FC| > 0.5 and an adjusted *p* value < 0.05. We visualized the results of these analyses with heat maps and volcano plots. To find common DEGs, we intersected the gene lists from both datasets using a Venn diagram, which yielded the subset of DEGs associated with both TED and GD.

### 2.3. Identification of Oxidative Stress–Related DEGs Related to TED and GD

From the GeneCards database, we identified 2598 oxidative stress–related genes using a relevance score greater than 1.5. Oxidative stress–related DEGs associated with TED and GD were determined by intersecting the TED/GD‐related DEGs with the oxidative stress gene set.

### 2.4. Enrichment Analyses of Oxidative Stress–Related DEGs Related to TED and GD

Kyoto Encyclopedia of Genes and Genomes (KEGG) and Gene Ontology (GO) pathway enrichment analyses were conducted on the oxidative stress–related DEGs to identify associated biological functions and pathways, using a significance threshold of *p* < 0.05.

### 2.5. Identification of Oxidative Stress–Related Hub DEGs Related to TED and GD

We applied LASSO and RF techniques to identify key diagnostic genes. A regression model was constructed in R using the “glmnet” package, with the penalty coefficient optimized via 10‐fold cross‐validation. The RF model was built with the “randomForest” package, from which the 10 most important variables were selected. The intersection of results from LASSO and RF analyses across both datasets was analyzed using a Venn diagram. A PPI network was constructed in the STRING database (https://string-db.org/) using a high‐confidence interaction score threshold of 0.7 and subsequently visualized with Cytoscape (Version 3.9.1) [14]. Hub genes were defined as those exhibiting the highest connectivity within the PPI network. The diagnostic performance of these hub genes was evaluated using ROC curves, which also facilitated a comparison of their expression between normal and disease groups across both datasets.

### 2.6. Nomogram Development Based on Diagnostic Biomarkers

Nomograms were constructed with the R package “rms” to evaluate the diagnostic utility of the oxidative stress–related hub DEGs. The predictive accuracy of these models was subsequently validated using ROC and calibration curves.

### 2.7. Immune Infiltration Analyses of TED and GD

**FIGURE 1:**
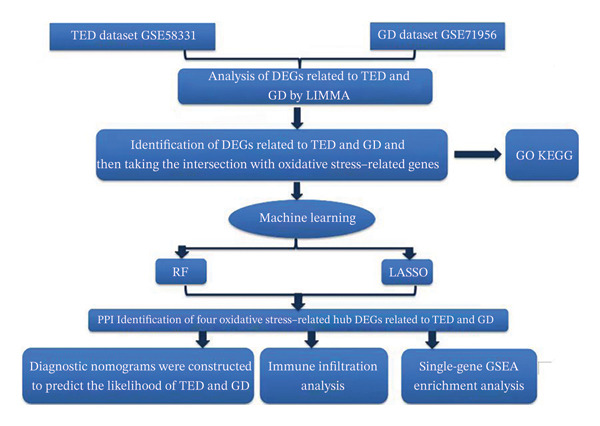
Flowchart of the study design.

The CIBERSORT tool quantified immune cell infiltration across 22 distinct immune cell types. We then assessed the correlations between the oxidative stress–related hub DEGs and these immune cell populations, presenting the results in heat maps. Finally, gene set enrichment analysis (GSEA) elucidated the biological functions associated with each hub gene (see Figure 1).

## 3. Results

### 3.1. Identification of DEGs Related to TED and GD

In the GSE58331 dataset, 651 DEGs were identified, including 135 upregulated and 516 downregulated genes. The GSE71956 dataset contained 1328 DEGs, with 483 upregulated and 845 downregulated. Volcano plots depict these DEGs, with upregulated genes shown in red and downregulated genes in blue (Figures 2(a) and 2(c)). Heat maps present the 10 most upregulated and downregulated genes from each dataset (Figures 2(b) and 2(d)). A Venn diagram analysis identified 58 DEGs common to both TED and GD (Figure 3(a)).

FIGURE 2Identification of differentially expressed genes (DEGs). DEG heat maps and volcano plots for the (a, b) GSE58331 and (c, d) GSE71956 datasets.

**Figure:**
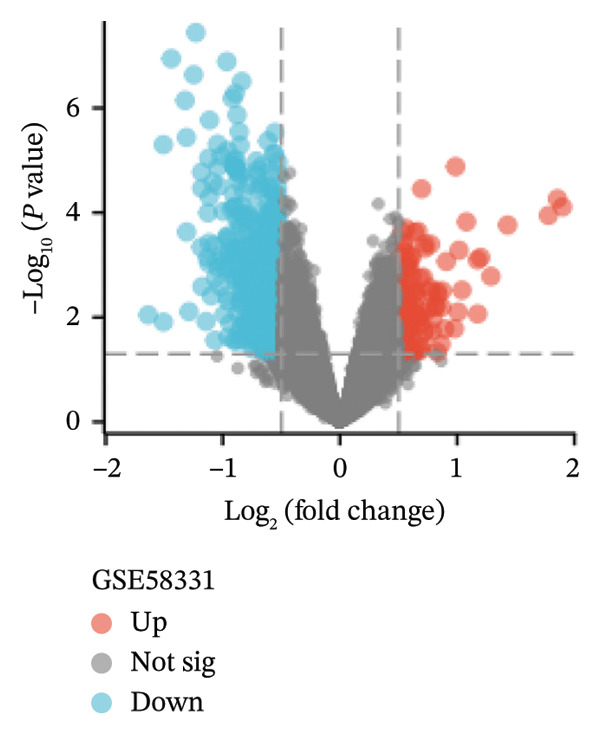
(a)

**Figure:**
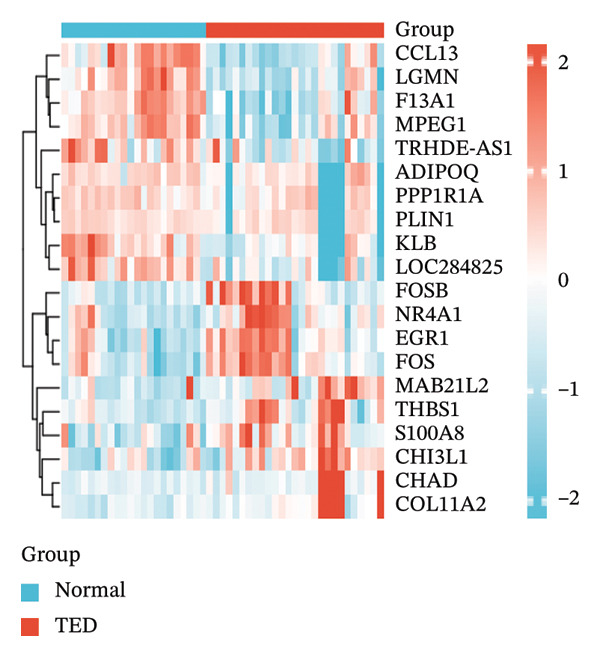
(b)

**Figure:**
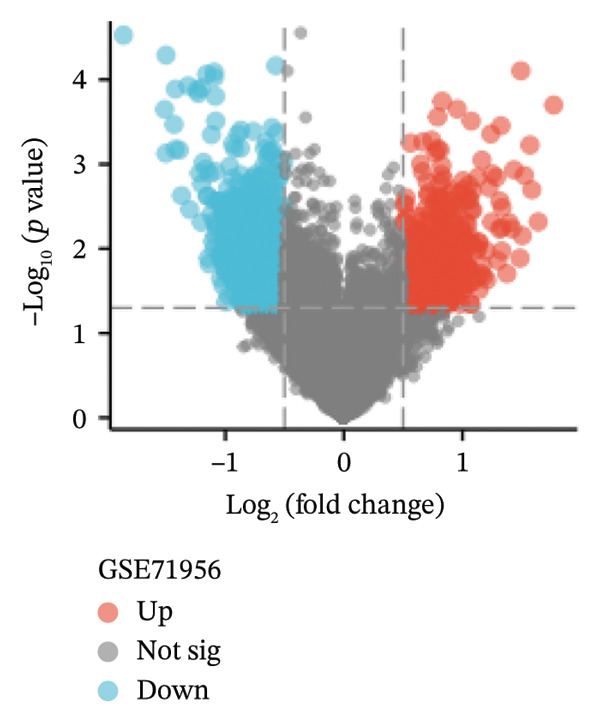
(c)

**Figure:**
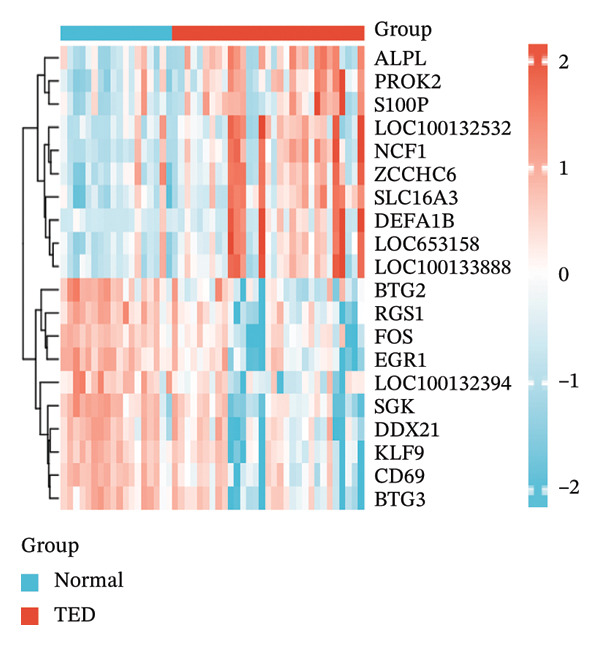
(d)

FIGURE 3Identification of oxidative stress–related DEGs and functional enrichment between TED and GD. (a) Venn diagram of the DEGs. (b) Venn diagram of the oxidative stress–related DEGs. (c) GO enrichment analyses of oxidative stress–related DEGs. (d) KEGG enrichment analyses of oxidative stress–related DEGs.

**Figure:**
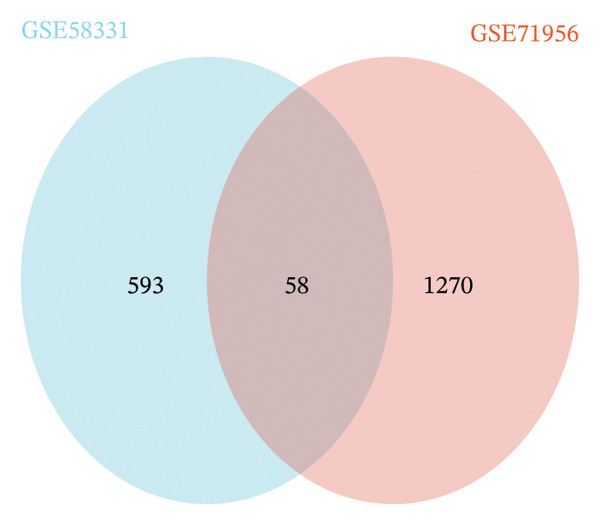
(a)

**Figure:**
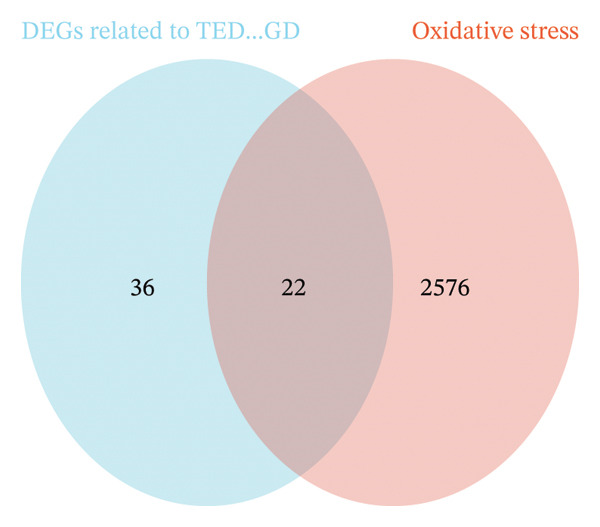
(b)

**Figure:**
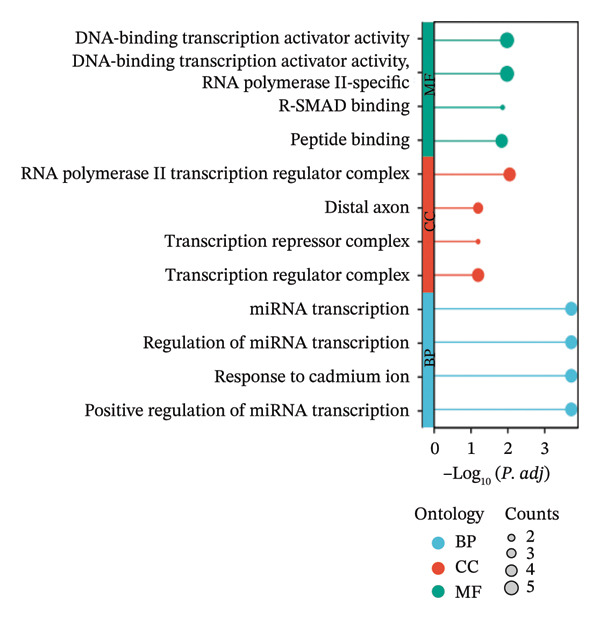
(c)

**Figure:**
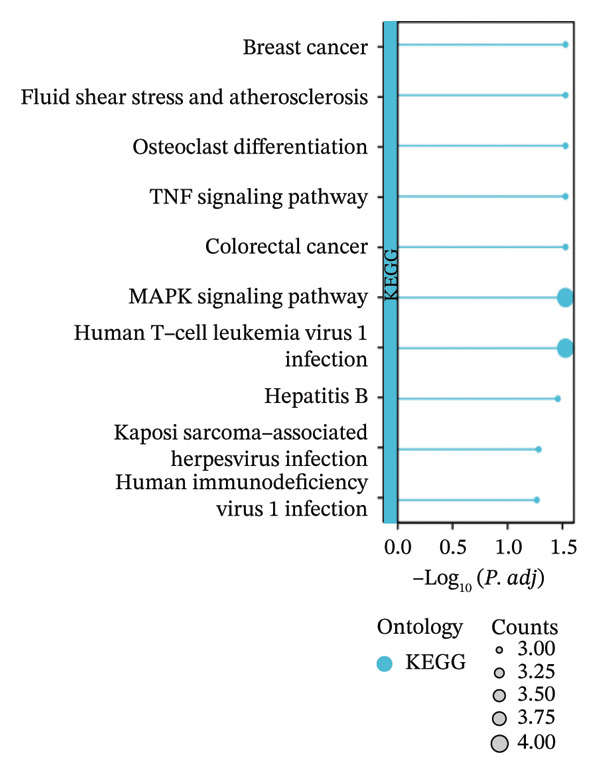
(d)

### 3.2. Identification of Oxidative Stress–Related DEGs Related to TED and GD

The intersection between TED/GD‐related DEGs and oxidative stress–related genes yielded 22 oxidative stress–related DEGs: EGR1, FOS, JUNB, MYC, DUSP1, CXCR4, MT2A, JUN, SFN, SGK1, UCP2, TPP1, ITM2B, BASP1, PRNP, TOMM20, RBM3, HEBP2, SEC11A, ANXA5, SETX, and ZMAT3 (Figure 3(b)). GO enrichment analysis indicated that these genes were primarily associated with miRNA transcription and its regulation (biological process), the RNA polymerase II transcription regulator complex (cellular component), and DNA‐binding transcription activator activity (molecular function) (Figure 3(c)). KEGG analysis showed enrichment in the MAPK signaling pathway and human T‐cell leukemia virus 1 infection (Figure 3(d)).

### 3.3. Screening Oxidative Stress–Related Hub DEGs Related to TED and GD and Their Diagnostic Values

LASSO analysis identified 11 relevant genes in TED and 6 in GD (Figures 4(a) and 4(b)), while the RF algorithm selected the top 10 genes for each condition (Figures 4(c) and 4(d)). The intersection of results from both methods yielded 11 oxidative stress–related DEGs: EGR1, FOS, JUNB, DUSP1, MT2A, SFN, UCP2, TPP1, RBM3, SEC11A, and ZMAT3 (Figure 4(e)). A PPI network constructed using the STRING database contained 4 nodes and 5 edges (Figure 4(f)), from which four hub genes (DUSP1, EGR1, FOS, and JUNB) were identified. ROC analysis demonstrated diagnostic value for all four hub genes, with the area under the curve (AUC) values exceeding 0.678 (Figure 4(g)). These genes were upregulated in the GSE58331 dataset (Figures 5(a), 5(b), 5(c), and 5(d)) and downregulated in the GSE71956 dataset (Figures 5(e), 5(f), 5(g), and 5(h)).

FIGURE 4Screening of oxidative stress–related hub genes and evaluation of their diagnostic values. (a) LASSO analysis for screening oxidative stress–related hub genes in GSE58331. (b) LASSO analysis for screening oxidative stress–related hub genes in GSE71956. (c) Identification of oxidative stress–related hub genes according to the importance of variables by random forest (RF) analysis of GSE58331. (d) Identification of oxidative stress–related hub genes according to the importance of variables by random forest (RF) analysis of GSE71956. (e) Venn diagram of the 11 common oxidative stress–related genes between the two algorithms. (f) The PPI network of 4 oxidative stress–related hub genes. (g) Receiver operating characteristic (ROC) curves of the 4 hub genes to assess their diagnostic values in the GSE58331 and GSE71956 datasets.

**Figure:**
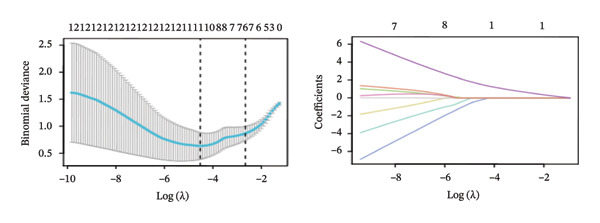
(a)

**Figure:**
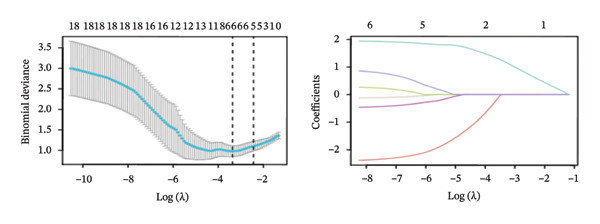
(b)

**Figure:**
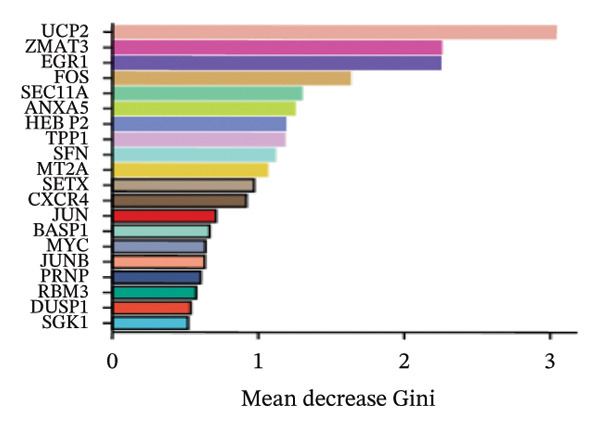
(c)

**Figure:**
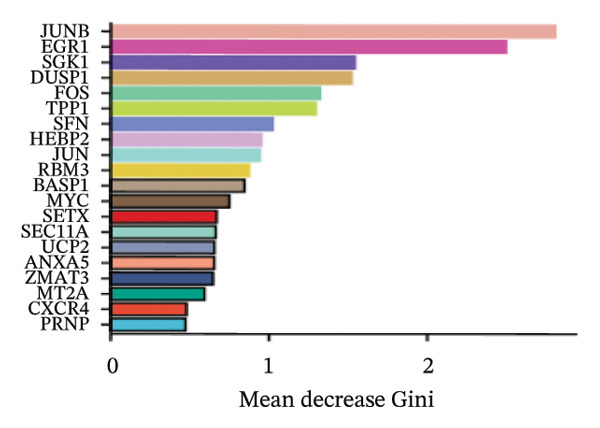
(d)

**Figure:**
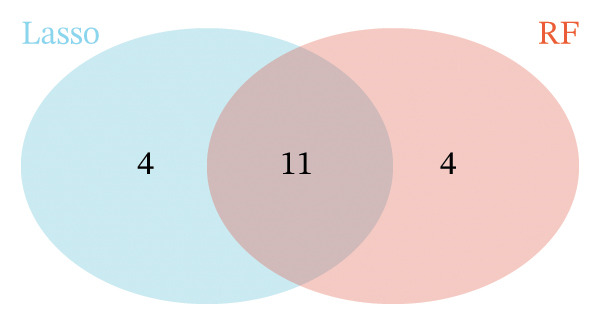
(e)

**Figure:**
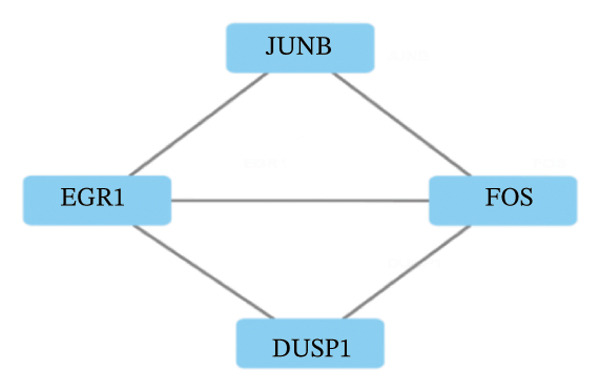
(f)

**Figure:**
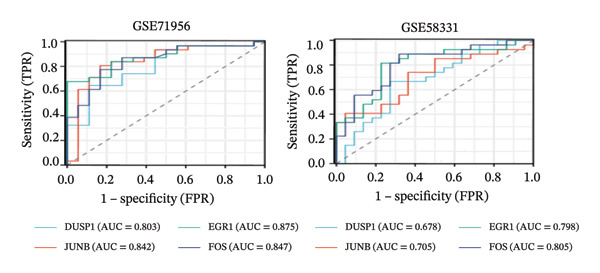
(g)

FIGURE 5Expression levels of the 4 hub genes in the GSE58331 and GSE71956 datasets. (a) Expression level of DUSP1 in GSE58331. (b) Expression level of EGR1 in GSE58331. (c) Expression level of JUNB in GSE58331. (d) Expression level of FOS in GSE58331. (e) Expression level of DUSP1 in GSE71956. (f) Expression level of EGR1 in GSE71956. (g) Expression level of JUNB in GSE71956. (h) Expression level of FOS in GSE71956. ^∗^ *p* < 0.05; ^∗∗^ *p* < 0.01; ^∗∗∗^ *p* < 0.001.

**Figure:**
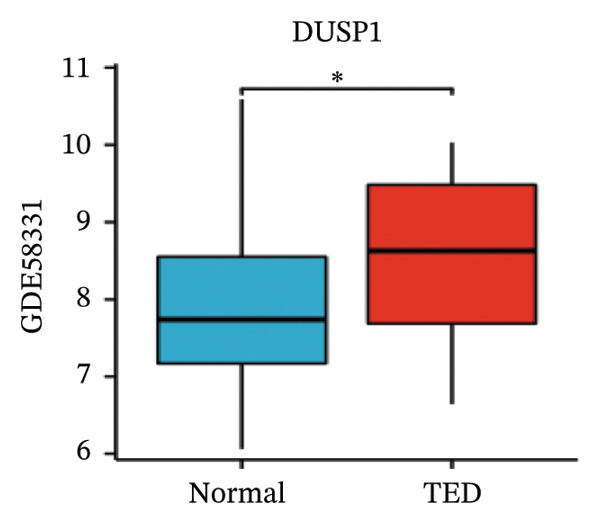
(a)

**Figure:**
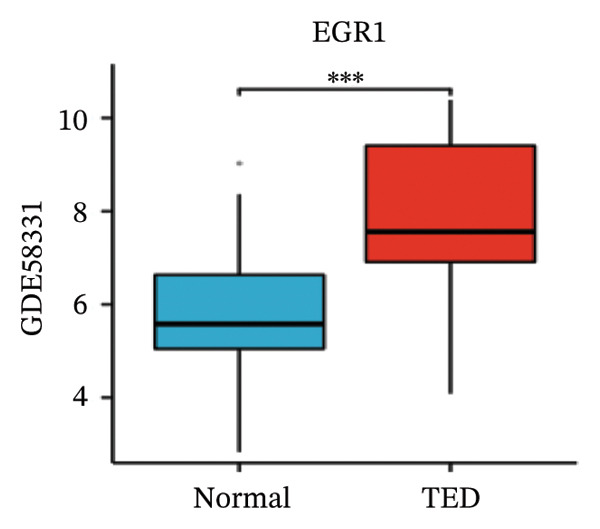
(b)

**Figure:**
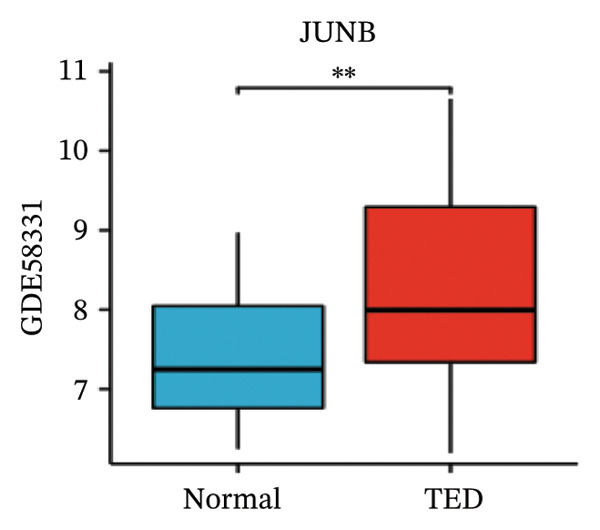
(c)

**Figure:**
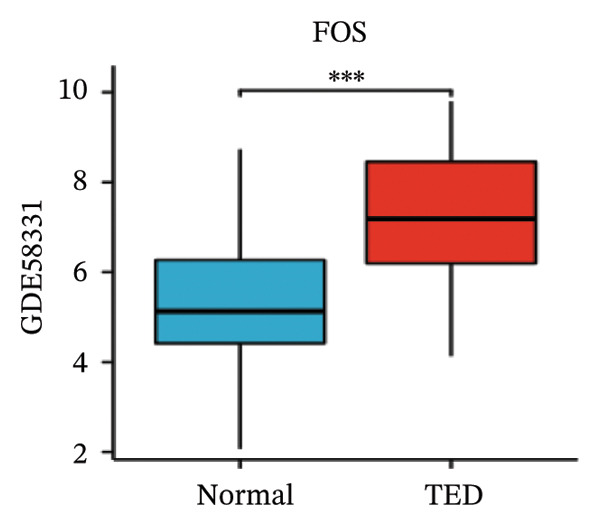
(d)

**Figure:**
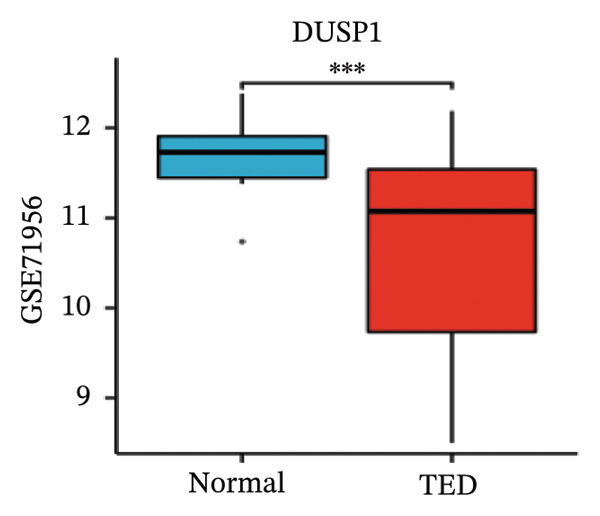
(e)

**Figure:**
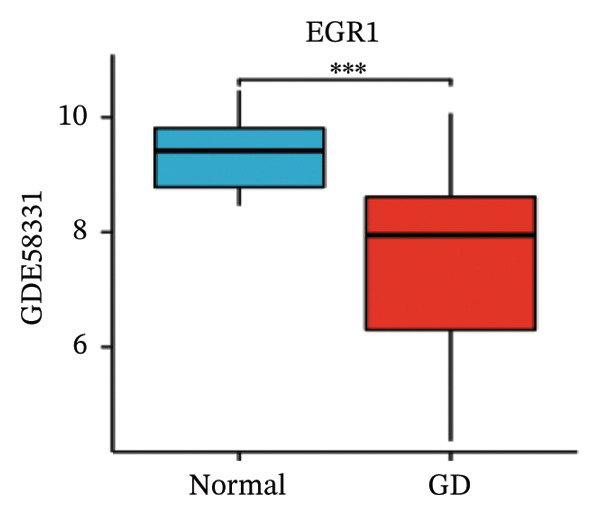
(f)

**Figure:**
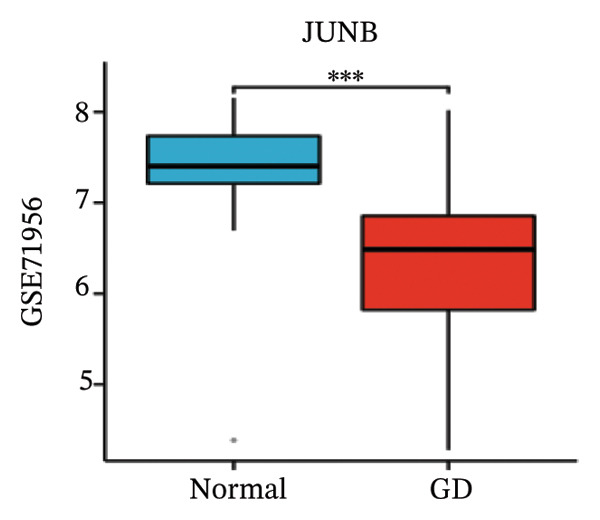
(g)

**Figure:**
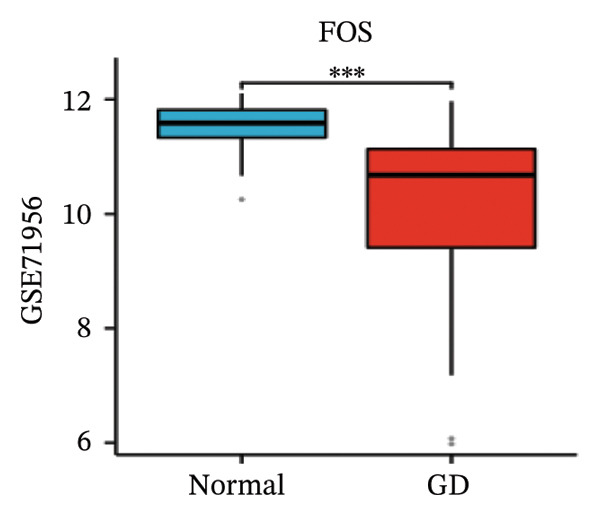
(h)

### 3.4. Nomogram Development Based on Diagnostic Biomarkers

Nomogram models based on the four hub genes were developed (Figures 6(a) and 6(d)). Calibration curves indicated close alignment with the ideal line, reflecting good model performance (Figures 6(b) and 6(e)). The model reliability was confirmed with the AUC values, which were 0.845 and 0.898 in two datasets (Figures 6(c) and 6(f)).

FIGURE 6Development of the diagnostic nomogram model. (a) Nomogram predicting the probability of TED. (b) Calibration curves of the TED risk models. (c) ROC curve of the TED risk model. (d) Nomogram predicting the probability of GD. (e) Calibration curves of the GD risk models. (f) ROC curve of the GD risk model.

**Figure:**
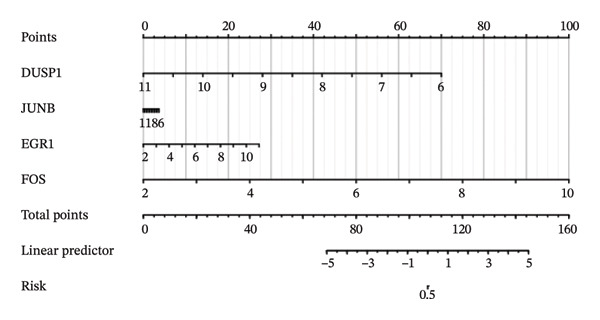
(a)

**Figure:**
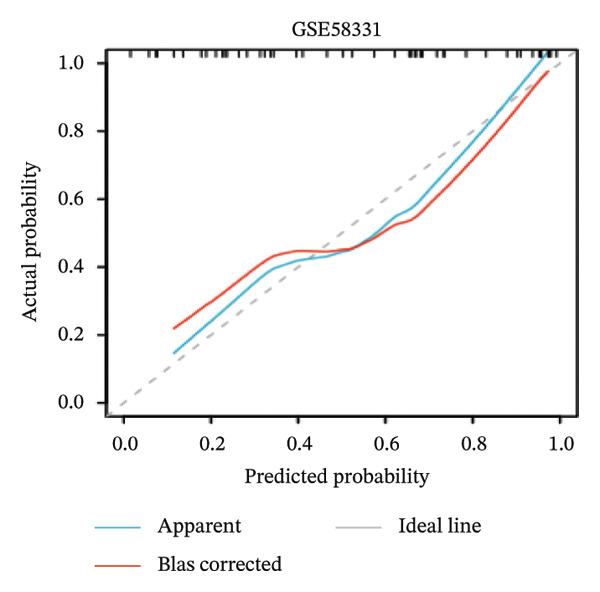
(b)

**Figure:**
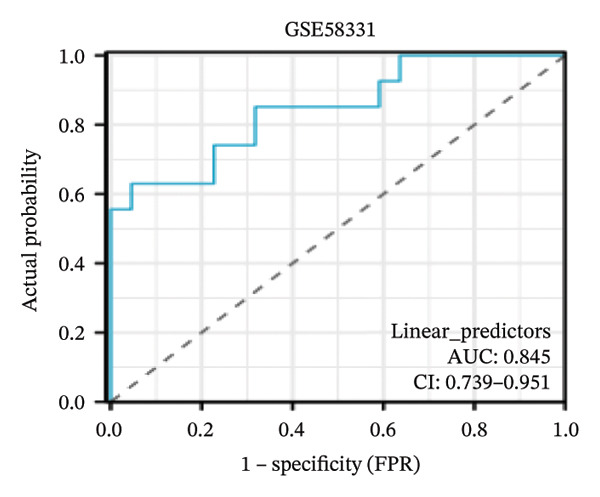
(c)

**Figure:**
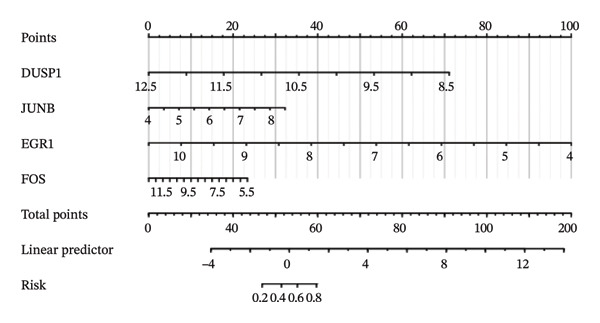
(d)

**Figure:**
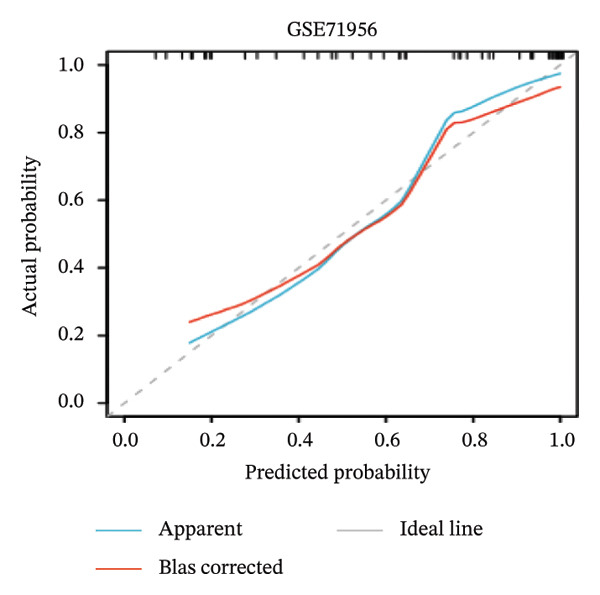
(e)

**Figure:**
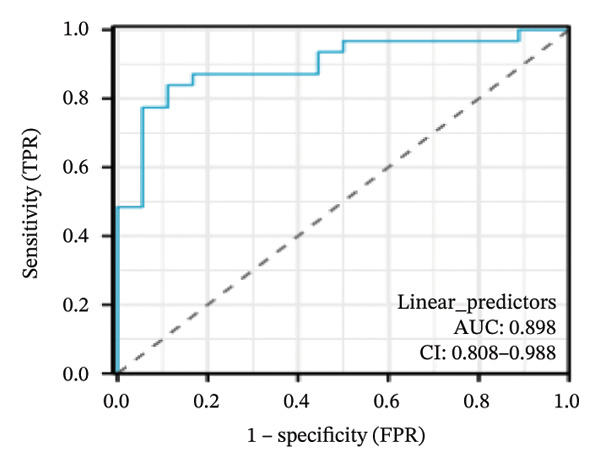
(f)

### 3.5. Immune Infiltration Analyses

CIBERSORT analysis revealed increased infiltrations of plasma cells, T follicular helper cells, macrophages M0, M1, and M2, monocytes, and resting and activated mast cells in the TED (Figure 7(a)). In GD, resting NK cells were elevated, while naïve B cells and activated dendritic cells were reduced (Figure 7(b)). The four hub genes showed strong associations with multiple immune cell types (Figures 7(c) and 7(d)). Single‐gene GSEA highlighted known pathways enriched for each hub gene, including DUSP1, EGR1, FOS, and JUNB in TED and GD tissues, suggesting their potential roles in disease pathogenesis (Supporting Figure A–H).

FIGURE 7Immune cell infiltration analyses in TED and GD. (a) Boxplot showing the comparison of 22 kinds of immune cells between TED and the normal group. (b) Boxplot showing the comparison of 22 kinds of immune cells between GD and the normal group. (c) Heat map representing the associations of the differentially infiltrated immune cells with the 4 hub genes in TED for the threshold of *p* < 0.05; ^∗^ *p* < 0.05; ^∗∗^ *p* < 0.01; and ^∗∗∗^ *p* < 0.001. (d) Heat map representing the associations of the differentially infiltrated immune cells with the 4 hub genes in GD for the threshold of *p* < 0.05; ^∗^ *p* < 0.05; ^∗∗^ *p* < 0.01; and ^∗∗∗^ *p* < 0.001.

**Figure:**
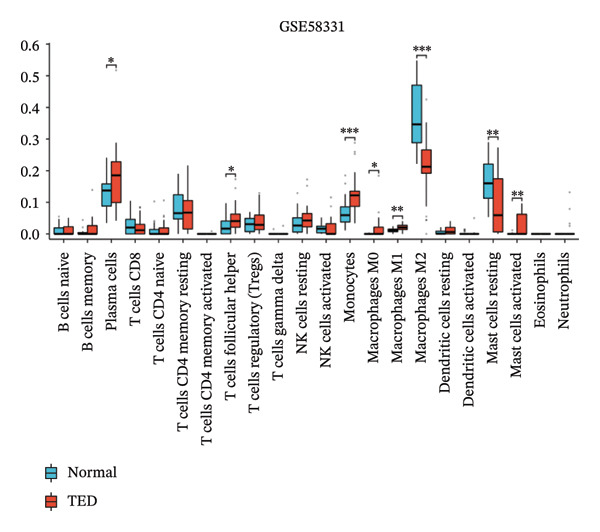
(a)

**Figure:**
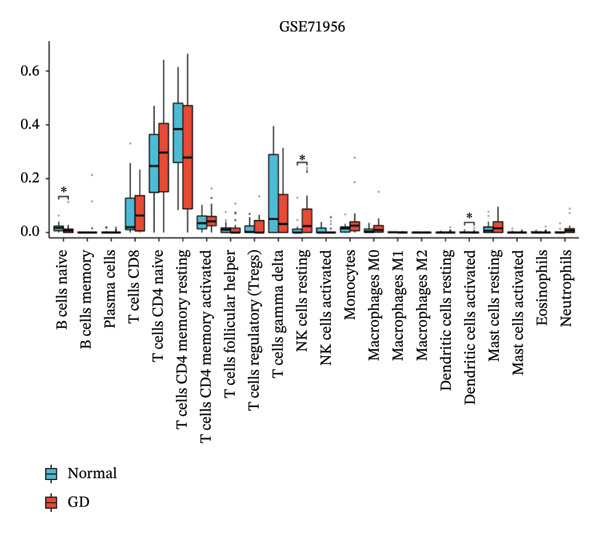
(b)

**Figure:**
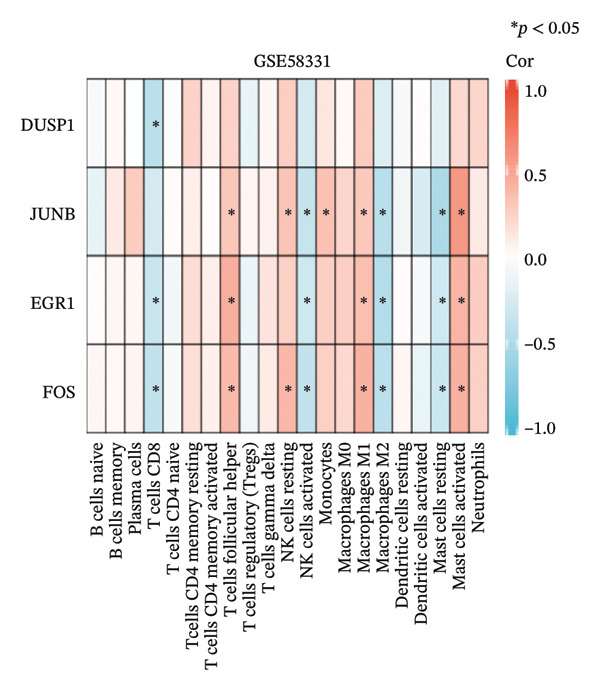
(c)

**Figure:**
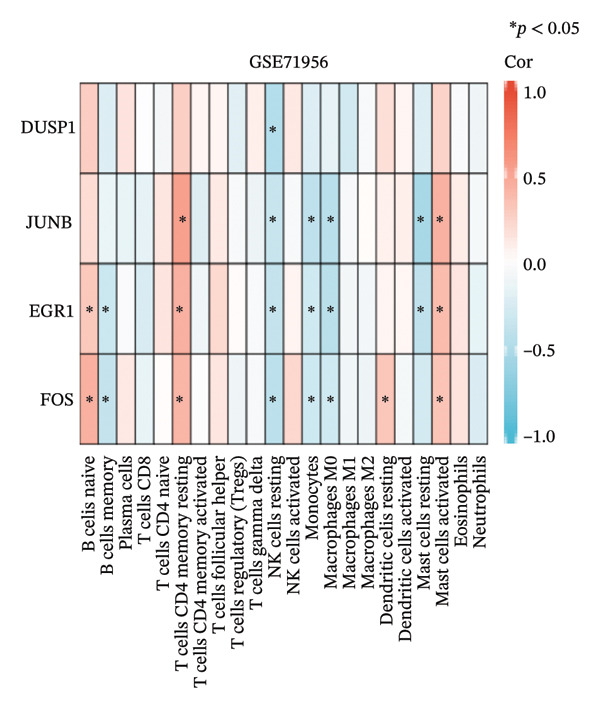
(d)

## 4. Discussion

In this study, the oxidative stress–related genes central to TED and GD were identified by analyzing GEO‐derived DEGs. Enrichment analysis of the intersection between oxidative stress–related genes and these DEGs revealed key biological activities and pathways. Critical diagnostic genes were identified using LASSO and RF algorithms, and hub genes were subsequently screened via PPI network analysis. Diagnostic nomograms were constructed, and immune infiltration patterns were evaluated. These results advance the understanding of the molecular mechanisms in TED and GD and could inform future diagnostic and therapeutic approaches.

KEGG pathway analysis revealed that oxidative stress–related DEGs in TED and GD were enriched in the MAPK signaling pathway and human T‐cell leukemia virus 1 infection. The MAPK signaling pathway, a critical intracellular cascade, regulates cell proliferation, differentiation, and survival [15]. Its dysregulation is implicated in numerous diseases; in TED and GD, alterations in this pathway may contribute to pathogenesis by perturbing thyroid cell function and immune responses. While human T‐cell leukemia virus 1 infection is an established etiological factor in specific leukemias [16], its direct role in TED and GD is not yet defined. The enriched genes associated with this pathway, however, may participate in immune dysregulation or other mechanisms relevant to disease. Additional pathways were also identified, though their specific roles and interactions in disease progression require further clarification. A detailed understanding of these molecular mechanisms is crucial for developing new therapeutic approaches.

The LASSO algorithm identified 11 oxidative stress–related DEGs in TED and 6 in GD. Subsequently, the RF algorithm prioritized the top 10 genes for each condition. The intersection of these results yielded 11 key DEGs: EGR1, FOS, JUNB, DUSP1, MT2A, SFN, UCP2, TPP1, RBM3, SEC11A, and ZMAT3. A PPI network analysis then identified four hub genes: EGR1, FOS, JUNB, and DUSP1. ROC analysis confirmed that all hub genes had AUCs exceeding 0.678; they were upregulated in the GSE58331 dataset but downregulated in GSE71956. A nomogram model incorporating these four genes demonstrated excellent diagnostic performance, with AUCs of 0.845 and 0.898 and good calibration. These findings indicate that the identified hub genes may be functionally important in the pathogenesis of TED and GD, while the nomogram represents a potential diagnostic tool. Further research studies are necessary to validate these results and elucidate the underlying mechanisms.

The four hub genes identified in this study—DUSP1, EGR1, FOS, and JUNB—play crucial roles in cellular stress responses and immune regulation. DUSP1, a dual‐specificity phosphatase, negatively regulates MAPK signaling pathways, which are activated in both TED and GD [17, 18]. The upregulation of DUSP1 in TED tissues may represent a feedback mechanism aimed at controlling excessive inflammation. EGR1 is an immediate‐early gene that responds to diverse stress stimuli and regulates the transcription of genes involved in cell growth and differentiation [19]. Dysregulation of EGR1 in TED and GD likely contributes to tissue remodeling and fibrosis. FOS, a component of the AP‐1 transcription factor complex, mediates cellular responses to stress and cytokines. AP‐1 signaling is implicated in the pathogenesis of autoimmune thyroid diseases [20–22], and our findings further support its role in TED and GD. JUNB, another component of the AP‐1 complex, modulates immune responses and may influence the infiltration of immune cells observed in TED and GD tissues [23, 24]. The coordinated dysregulation of these genes suggests that they may be involved in shared pathogenic mechanisms underlying TED and GD. The roles of DUSP1, EGR1, FOS, and JUNB in stress responses and immune regulation highlight their potential importance in the pathogenesis of TED and GD. Understanding the functions of these genes and their interactions could elucidate the conserved mechanisms underlying these conditions and ultimately guide the development of novel therapies.

Using the innovative CIBERSORT algorithm, we delved into the intricate world of immune cell infiltration within cohorts affected by TED and GD, as documented in the datasets GSE58331 and GSE71956. Our analysis revealed remarkable increases in an array of immune cell types in TED, including plasma cells, T cells follicular helper, mast cells (both resting and activated), monocytes, and macrophages (M0, M1, and M2). Conversely, in GD, levels of resting NK cells were elevated, while naïve B cells and activated dendritic cells showed a decrease. Of particular interest was the correlation between four key hub genes and the various immune cell types found in both conditions. For instance, the heightened presence of plasma cells in TED suggested an intensified humoral immune response, with these cells being major players in antibody secretion [25]. Similarly, the increased levels of follicular helper T cells indicated an active humoral response, supporting B cell activation and antibody production [26]. The surge in monocytes and macrophages in TED hinted at ongoing inflammation and potential tissue damage, as these cells are known to participate in inflammation and tissue remodeling processes [27]. Mast cells, implicated in allergic and inflammatory responses, may also contribute to TED pathogenesis [28]. The findings in GD painted a different picture, with elevated resting NK cells pointing toward heightened innate immunity, crucial for immune surveillance and defense mechanisms [29]. Conversely, the reduced levels of naïve B cells and activated dendritic cells suggested a hampered adaptive immune response. The close relationship between the identified hub genes and immune cells raises intriguing questions about their potential regulatory roles in modulating immune responses that contribute to the pathogenesis of TED and GD. Further research is warranted to unravel the precise mechanisms underlying these interactions.

Oxidative stress contributes to TED pathogenesis through multiple mechanisms [30, 31]. ROS directly damage orbital tissues, provoke inflammation, and promote fibroblast proliferation, ultimately leading to the tissue remodeling and fibrosis characteristic of TED. Studies have consistently shown that patients with TED exhibit elevated levels of oxidative stress markers, including 8‐hydroxy‐2′‐deoxyguanosine (8‐OHdG) and malondialdehyde (MDA), while the activity of antioxidant enzymes such as GPx and SOD is reduced [29]. Various studies have delved into the potential of antioxidants in treating TED, with antioxidants such as vitamin E, N‐acetylcysteine (NAC), and selenium showing promise in reducing oxidative stress and inflammation in experimental models and small clinical trials [32, 33]. For example, selenium supplementation has been linked to enhanced quality of life and reduced eye complications in mild TED patients. Similarly, NAC has exhibited protective effects against oxidative stress in orbital fibroblasts. The identification of hub genes related to oxidative stress offers possible molecular targets that could be influenced by antioxidant therapies, opening up new possibilities for the development of treatments for TED.

Our research delves into a thorough examination of oxidative stress–related genes in both TED and GD, a unique approach not previously seen in existing literature [31, 34]. By utilizing a comprehensive bioinformatics method, we were able to identify a robust set of DEGs associated with oxidative stress, validating their importance through enrichment analyses and advanced machine learning techniques. A key novel contribution of our work is the exploration of how these genes interact with immune cell infiltration, offering valuable insights into the underlying mechanisms of these conditions. This novel research not only sheds light on the molecular pathways involved in TED and GD but also opens up possibilities for the development of innovative diagnostic and therapeutic approaches. While our findings present promising potential biomarkers, further research is necessary to confirm the results and assess their clinical implications. Our work represents a crucial step toward enhancing our understanding of these diseases and improving patient outcomes in the future.

Our study could not directly compare oxidative stress–related gene expression between GD patients with and without TED due to the lack of well‐annotated public datasets. Nevertheless, our findings provide valuable indirect insights. The varying expression patterns of key genes—elevated in TED orbital tissues but reduced in GD T cells—point toward tissue‐specific disease mechanisms, potentially reflecting local inflammation as opposed to immune exhaustion in the periphery. These discrepancies align with the known functions of DUSP1, EGR1, FOS, and JUNB in regulating oxidative stress and the immune system [35–37]. We stress the importance of future studies adopting prospective cohort designs to elucidate these differences and investigate the therapeutic benefits of targeting these pathways.

The present study employed comprehensive bioinformatics analyses to identify oxidative stress–related hub DEGs related to TED and GD. It is essential to acknowledge that this research solely relies on computational methods and lacks experimental verification. To validate the findings, further in vitro and in vivo experiments such as qPCR, western blot, and immunohistochemistry on patient samples are imperative. The data used were sourced from a single database, potentially leading to bias. Moreover, the reliance on publicly available datasets necessitates additional validation through functional experiments. Moving forward, it is crucial for subsequent studies to address these shortcomings by leveraging multiple datasets and performing functional experiments to confirm the roles of the identified hub genes in the development of TED and GD. By doing so, a more comprehensive understanding of the pathogenesis of these diseases can be achieved.

## 5. Conclusion

Our study identifies candidate biomarkers and therapeutic targets for TED and GD, offering preliminary insights into their molecular mechanisms. These findings necessitate experimental validation to explore their potential clinical applications.

**Nomenclature**
AUC: The area under the curve
DEGs: Differentially expressed genes
GD: Graves’ disease
GEO database: Gene Expression Omnibus database
GO: Gene Ontology
GSEA: Gene set enrichment analysis
KEGG: Kyoto Encyclopedia of Genes and Genomes
LASSO: Least absolute shrinkage and selection operator
PPI: Protein–protein interaction
RF: Random forest
ROC: Receiver operating characteristic
TED: Thyroid eye disease

## Author Contributions

W.Z.: conceptualization, methodology, software, and writing–original draft preparation. Q.H.: data curation. J.L.: visualization, supervision, and writing–reviewing and editing.

## Funding

There was not any financial support by other institutions.

## Disclosure

All authors have read and approved the manuscript.

## Ethics Statement

Since our research does not involve experiments on human participants or the use of personal or sensitive data, there was no requirement for institutional ethical review or approval.

## Consent

The authors have nothing to report.

## Conflicts of Interest

The authors declare no conflicts of interest.

## Data Availability

The authors have nothing to report.
